# Sexual and reproductive health and human rights of women living with HIV: a global community survey

**DOI:** 10.2471/BLT.14.150912

**Published:** 2016-02-12

**Authors:** Manjulaa Narasimhan, Luisa Orza, Alice Welbourn, Susan Bewley, Tyler Crone, Marijo Vazquez

**Affiliations:** a Department of Reproductive Health and Research, World Health Organization, 20 avenue Appia, CH-1211 Geneva 27, Switzerland.; b Athena Network, London, England.; c Salamander Trust, London, England.; d Women’s Health Academic Centre, King’s College London, London, England.; e ATHENA Network, Seattle, United States of America.; f Salamander Associate, Barcelona, Spain.

## Abstract

**Objective:**

To determine the sexual and reproductive health priorities of women living with human immunodeficiency virus (HIV) and to allow the values and preferences of such women to be considered in the development of new guidelines.

**Methods:**

A core team created a global reference group of 14 women living with HIV and together they developed a global community online survey. The survey, which contained mandatory and optional questions, was based on an appreciative enquiry approach in which the life-cycle experiences of women living with HIV were investigated. The same set of questions was also used in focus group discussions led by the global reference group.

**Findings:**

The study covered 945 women (832 in the survey and 113 in the focus groups) aged 15–72 years in 94 countries. Among the respondents to the optional survey questions, 89.0% (427/480) feared or had experienced gender-based violence, 56.7% (177/312) had had an unplanned pregnancy, 72.3% (227/314) had received advice on safe conception and 58.8% (489/832) had suffered poor mental health after they had discovered their HIV-positive status.

**Conclusion:**

The sexual and reproductive health needs and rights of women living with HIV are complex and require a stronger response from the health sector. The online survey placed the voices of women living with HIV at the start of the development of new global guidelines. Although not possible in some contexts and populations, a similar approach would merit replication in the development of guidelines for many other health considerations.

## Résumé

**Objectif:**

Déterminer les priorités en matière de santé sexuelle et génésique des femmes vivant avec le virus de l'immunodéficience humaine (VIH) et permettre la prise en considération des valeurs et préférences de ces femmes lors de l'élaboration de nouvelles lignes directrices.

**Méthodes:**

Une équipe de base a formé un groupe de référence mondial, composé de 14 femmes vivant avec le VIH, avec lequel elle a préparé une enquête communautaire en ligne à l'échelle mondiale. Cette enquête, qui comportait des questions obligatoires et facultatives, s'appuyait sur une démarche appréciative qui a permis d'examiner l'expérience de vie des femmes vivant avec le VIH. La même série de questions a été utilisée lors de réunions de groupes de discussion animées par le groupe de référence mondial.

**Résultats:**

L'étude a pris en compte 945 femmes (832 qui ont répondu à l'enquête et 113 dans les groupes de discussion) âgées de 15 à 72 ans dans 94 pays. Parmi les femmes ayant répondu aux questions facultatives de l'enquête, 89,0% (427/480) craignaient ou avaient déjà subi des violences sexistes, 56,7% (177/312) avaient eu une grossesse non désirée, 72,3% (227/314) avaient reçu des conseils sur la conception en toute sécurité et 58,8% (489/832) avaient connu des problèmes de santé mentale après avoir appris qu'elles étaient séropositives.

**Conclusion:**

Les droits et les besoins des femmes vivant avec le VIH en matière de santé sexuelle et génésique sont complexes et nécessitent une réponse plus forte du secteur de la santé. Cette enquête en ligne a permis aux femmes vivant avec le VIH de faire entendre leur voix pour l'élaboration de nouvelles lignes directrices mondiales. Bien que cela ne soit pas possible dans certains contextes et certaines populations, une démarche similaire mériterait d'être appliquée à l'élaboration de lignes directrices concernant de nombreuses autres questions de santé.

## Resumen

**Objetivo:**

Determinar las prioridades de salud sexual y reproductiva de mujeres que viven con el virus de la inmunodeficiencia humana (VIH) y permitir la consideración de los valores y preferencias de dichas mujeres en el desarrollo de nuevas directrices.

**Métodos:**

Un equipo básico ha creado un grupo de referencia mundial de 14 mujeres que viven con el VIH, las cuales, juntas, han desarrollado una encuesta comunitaria en línea a nivel mundial. La encuesta, que contiene preguntas obligatorias y opcionales, se basó en un enfoque de investigación apreciativa en el cual se han estudiado las experiencias vitales de mujeres que viven con el VIH. También se utilizó el mismo conjunto de preguntas en grupos de discusión dirigidos por el grupo de referencia mundial.

**Resultados:**

El estudio cubrió 945 mujeres (832 en la encuesta y 113 en los grupos) de 94 países de edades comprendidas entre los 15 y los 72 años. Entre las encuestadas que respondieron las preguntas opcionales, el 89,0% (427/480) había temido o experimentado violencia de género, el 56,7% (177/312) había sufrido un embarazo no deseado, el 72,3% (227/314) había recibido asesoría sobre la concepción segura y el 58,8% (489/832) había sufrido problemas de salud mental tras descubrir que son VIH positivas.

**Conclusión:**

Las necesidades de salud sexual y reproductiva y los derechos de las mujeres que viven con el VIH son complejos y requieren una mayor respuesta por parte del sector sanitario. La encuesta en línea dio voz a mujeres que viven con el VIH al principio del desarrollo de nuevas directrices mundiales. Aunque en algunos contextos y poblaciones resulta imposible, es necesario adoptar un enfoque similar en el desarrollo de directrices por muchos otros motivos sanitarios.

## ملخص

**الغرض:**

تحديد أولويات الصحة الجنسية والإنجابية لدى النساء المتعايشات مع فيروس نقص المناعة البشري (HIV) والسماح بمراعاة القيم التي تؤمن بها هؤلاء النساء وتفضيلاتهن عند وضع المبادئ التوجيهية الجديدة.

**الطريقة:**

تولى فريق أساسي إعداد فريق مرجعي عالمي يتكون من 14 امرأة متعايشة مع فيروس نقص المناعة البشري (HIV) وتعاون الفريقان في إنشاء استطلاع للرأي عبر الإنترنت من خلال مجتمع عالمي. واعتمد استطلاع الرأي – الذي تضمن بعض الأسئلة الإجبارية وأخرى اختيارية – على أسلوب الاستقصاء الإيجابي الذي تم من خلاله البحث في تجارب النساء المتعايشات مع فيروس نقص المناعة البشري (HIV) في مراحل الحياة المختلفة. وتم أيضًا استخدام نفس مجموعة الأسئلة في النقاشات التي أدارها الفريق المرجعي العالمي فيما بين أعضاء مجموعة التركيز.

**النتائج:**

شملت الدراسة 946 امرأة (حيث تضمنت الدراسة 832 منهن بينما شملت مجموعات التركيز 113 منهن) من 94 دولة، وتراوحت أعمارهن بين 15 و72 سنة. واشتملت مجموعة المشاركات اللاتي أجبن على الأسئلة الاختيارية في استطلاع الرأي على 89.0‏% (‏427/480) ممن عانين من الخوف أو العنف ضد المرأة، و56.7‏% (177‏/312) ممن تعرضن للحمل دون تخطيط منهن، و72.3‏% (‏227/314) ممن تلقين النصح بشأن الحمل الآمن و58.8‏‏% (489‏/832) ممن عانين من تدهور الصحة العقلية بعد اكتشافهن حالة الإصابة بفيروس نقص المناعة البشري (HIV).

**الاستنتاج:**

تتسم الاحتياجات والحقوق المتعلقة بالصحة الجنسية والإنجابية التي تستحقها النساء المتعايشات مع فيروس نقص المناعة البشري (HIV) بالتعقيد ويتطلب الأمر استجابة أكثر قوة من جانب قطاع الصحة. وقد وضع استطلاع الرأي عبر الإنترنت أصوات النساء المتعايشات مع فيروس نقص المناعة البشري (HIV) في بداية مراحل وضع المبادئ التوجيهية العالمية الجديدة. ويجدر اتباع أسلوب منهجي مماثل أثناء وضع المبادئ التوجيهية المتعلقة بالعديد من الاعتبارات الصحية الأخرى، رغم عدم إمكانية تطبيق ذلك في ظل بعض الظروف والتركيبات السكانية.

## 摘要

**目的:**

旨在确定感染艾滋病毒 (HIV) 女性的性和生殖健康，并在制定新指南过程中考虑此类女性的价值和偏好。

**方法:**

核心团队创建一支由 14 名感染艾滋病毒女性组成的全球参照组，共同开展全球社区在线调查。本次调查采用肯定式询问法，包含一些必答和选答问题，从而调查感染艾滋病毒女性的生命周期体验。同一组问题同样用于全球参照组主导的重点小组讨论。

**结果:**

本次调查涉及 94 个国家中 945 名 15-72 岁的女性（参与调查的 832 名和重点小组的 113 名）。参与选答问题调查的受访者中，89.0% (427/480) 害怕或曾体验过性别暴力；56.7% (177/312) 曾经历了意外怀孕；72.3% (227/314) 获得过安全概念的建议及 58.8% (489/832) 在发现其 HIV 检测结果呈阳性后患上心理问题。

**结论:**

感染艾滋病毒女性的性和生殖健康需求及权利很复杂，需要卫生部门给予更有力的回应。本次在线调查在开始制定全球新指南时，评定感染艾滋病毒女性的声音。尽管这在有些环境和人口不可能实现，但是出于众多其他卫生因素考虑，制定指南时，值得采用类似方法。

## Резюме

**Цель:**

Определить приоритеты женщин, живущих с ВИЧ, в сфере сексуального и репродуктивного здоровья и создать возможности для того, чтобы их ценности и предпочтения были учтены при разработке новых руководящих принципов.

**Методы:**

Основная группа сформировала глобальную референтную группу из 14 женщин, живущих с ВИЧ, вместе с которой был подготовлен интернет-опрос мирового сообщества. В основу опроса, предполагающего как обязательные, так и дополнительные вопросы, был заложен поощрительный подход с целью изучения жизненного опыта женщин, живущих с ВИЧ. Глобальная референтная группа также проводила дискуссии в фокус-группах, в которых использовался тот же набор вопросов.

**Результаты:**

В исследовании приняли участие 945 женщин (832 опрошенных и 113 членов целевых групп) в возрасте от 15 до 72 лет в 94 странах. Среди лиц, ответивших на факультативные вопросы, 89,0% (427 из 480) опасаются гендерного насилия или сталкивались с ним, 56,7% (177 из 312) сталкивались с незапланированной беременностью, 72,3% (227 из 314) консультировались на тему безопасной контрацепции, у 58,8% (489 из 832) развивалось какое-либо психическое расстройство после того, как у них был диагностирован ВИЧ-положительный статус.

**Вывод:**

Потребности и права женщин, живущих с ВИЧ, в сфере сексуального и репродуктивного здоровья представляют собой сложную систему и требуют более значительных мер со стороны системы здравоохранения. Благодаря интернет-опросу голоса женщин, живущих с ВИЧ, получили приоритет при разработке новых руководящих принципов. Аналогичный подход стоит применять при разработке руководящих принципов для решения многих других задач здравоохранения, хотя при определенных условиях и для отдельных групп населения это не представляется возможным.

## Introduction

In the development of any of its global guidelines, the World Health Organization (WHO) places importance on the values and preferences of the population or individuals that could be affected by the recommendations made within the guidelines.^1^ WHO has guidelines on the care, treatment and support for women living with HIV and their children in resource-constrained settings,^2^ but these guidelines were published in 2006 and require updating. As an initial step in the updating process, WHO commissioned a global survey to listen to the voices of women living with HIV and determine these women’s sexual and reproductive health priorities. The main aim of the survey was to ensure that the values and preferences of women living with HIV would inform the guidelines from the very start of its development. The methods and key outcomes of the global survey are described and discussed below.

## Methods

As no single network is likely to represent the wide diversity of women living with HIV, the investigation was based on a survey tool that (i) had been developed by women living with HIV in collaboration with a range of organizations and (ii) reflected a broad range of expertise, geographical perspectives and key affected populations.^3^ A core team, which had been established to coordinate and lead the survey development, created a global reference group of women living with HIV. The 14 women in this reference group represented a wide range of national, regional and global organizations of women living with HIV – including adolescents and elderly women and transgender, lesbian, bisexual and other women who have sex with women – and a wide range of experiences – including perinatal transmission, sexual violence or rape, comorbidities of tuberculosis and/or hepatitis C, current or previous use of drugs and prison or detention.^4^

### Pre-survey consultation

In a pre-survey consultation based on a quality of life and dimensions of well-being exercise,^5^ each member of the global reference group explored how the material, physical, psychological, sexual and spiritual dimensions of her quality of life – including some deeply personal issues – affected her sexual and reproductive health and vice versa. The aim was to reveal the key sexual and reproductive health priorities in the lives of women living with HIV.

### Survey

Subsequently, the members of the global reference group and the core team worked together to develop a global online survey. The overarching human-rights-related priority issues that emerged from the comprehensive pre-survey consultation, formed the basis of the first – mandatory – section of the survey whereas other key priorities formed the basis of the other sections, all of which were optional. The optional sections related to (i) a healthy sex life, (ii) pregnancy and fertility, (iii) violence against women living with HIV, (iv) mental-health issues, (v) women living with HIV in all of their diversity, (vi) puberty, menstrual issues and menopause, (vii) HIV treatment and side effects and (viii) financial issues affecting access to services. The whole survey was based on an appreciative enquiry life-course approach^6^ that included both quantitative and qualitative aspects of the lives and experiences of women living with HIV.^7^ The survey platform was designed not only to enable the surveyed women to tell their stories and reflect on all stages of their lives, but also to establish the values–preferences input for the development of new WHO guidelines.

The content of the survey tool was developed following broad stakeholder outreach, several teleconferences and meetings and a consultation with the Joint United Nations Programme on HIV/AIDS’s dialogue platform of women living with HIV, and other United Nations partners. By following existing recommendations for mixed methods research in the health sciences^8^ and using Survey Monkey software (Survey Monkey, Palo Alto, United States of America), the tool was adjusted and finalized over a period of three months. The survey tool began with background information on the purpose, aims, content and background of the survey, a list of key definitions and a description of how the survey findings would be used. A woman invited to complete the online survey had to answer “yes” to the initial question – i.e. “Are you a woman living with HIV?”^3^ – before she could see and answer any further questions. As well as quantitative, closed-answer questions, all of the survey’s nine sections included spaces in which respondents could elaborate on their answers – e.g. they could give details of their own experience, context and/or perceived barriers to accessing services and/or achieving rights. Most of the closed-answer questions were worded positively and either solution-oriented or human-rights oriented^9^ but the sections exploring violence and mental health included questions about negative experiences. The survey included follow-up questions – either free-text or closed – about positive experiences and/or examples of best practice, sources of support, resilience and solution-oriented, rights-affirming recommendations.

Face-to-face focus group discussions using the same set of questions as the online survey were designed to run concurrently, for participants with no or limited computer access or literacy. It was hoped that these discussions and the online survey would create a safe virtual or physical space for women living with HIV to share their experiences. The survey tool, which was created in English, was translated, by volunteers, first into French, Russian and Spanish and subsequently into Bahasa Indonesian, Chinese and Portuguese. The translators were all women living with HIV or affected by the virus, who perceived the importance of this work. All considered themselves to be activists who were familiar with the language and content of the survey and were sensitive to using rights-based, gender-equitable and culturally sensitive language. Although a professional translator synthesized the French, Portuguese and Spanish responses, Google Translate software (Google, Mountain View, USA) was used to convert the Bahasa Indonesian, Chinese and Russian responses into English.

Focus group discussions in English, French and five further languages (Table 1) were facilitated by 10 women – including six women living with HIV – who were either members of the global reference group or women who already had established relationships with – and were trusted by – the participants. All of these facilitators worked professionally within the HIV, gender and human-rights sectors and had experience in conducting focus group discussions, workshops, training sessions and advocacy meetings. Most were already known to the core team and had been involved in previous related studies.^4^^,^^10^ The core team and facilitators adapted and amended an initial discussion guide to match the particular population of women involved in each discussion. The first focus group discussion, which was undertaken in Thailand, served as a pilot. Facilitators identified the key themes of each discussion in a short report. These reports were returned to a coordinator and shared with the core team. Any requests for clarification or more detail were collated and sent back to the facilitators. The findings were then used to supplement and enrich the qualitative data collected through the online survey, with particular reference to women in specific contexts. The online survey data were, however, the primary source used to articulate, shape and structure the main findings listed in the overall report.^4^

**Table 1:** Details of the focus group discussions held with 113 women living with HIV, in seven countries, 2014

Country	Language	Participants**^a^**
Ethiopia	Amharic	One discussion with 20 rural women living with HIV and one with 10 sex workers
Jamaica	English	Eight women aged 41–63 years living with HIV
Myanmar	Myanma bhasa	10 young female teenagers who were born with HIV
Nepal	Nepali	One discussion with 10 widows, aged 28–55 years, whose husbands were migrant workers, one with four sex workers aged 17–25 years, and one with five transgender women
Senegal	French and Wolof	One discussion with 20 sex workers aged 17–65 years and one with 10 women – some of whom were physically disabled
Thailand	Thai	Five women – two of whom were drug users
United Kingdom	English	11 migrant women – three of whom had been in prison or detention

HIV: human immunodeficiency virus.

^a^ Ages are shown only when they were recorded.

### Ethics

Ethical considerations were made in line with international guidelines on research on women living with HIV^11^ or domestic violence against women.^12^ Potential respondents who still wished to participate in the online survey after reading the introductory text were required to give their informed consent, by clicking on the relevant box, before they could proceed.

A potential participant in a focus group discussion gave her explicit written or verbal consent before the discussion began. Participants were told that they could drop out of the discussion at any time or choose not to answer any individual questions. Participants in the online survey were informed about how the data would be handled and that any identifying information would be removed if quotes were used. The name of each participant, the organization she belonged to – if any – and her within-country location were removed before the results were collated. Any paperwork that held such details was shredded.

### Timing and promotion

The online survey and focus group discussions took place between February and June of 2014 and were entirely managed by and for the community of women living with HIV. Survey announcements were disseminated widely by the global reference group and core team, women’s groups and support groups, on Facebook and Twitter and via the relevant United Nations partners, donor agencies and nongovernmental organizations.^10^ In addition, the global reference group and core team sent emails and made phone calls to individual women and activists with broad networks and to individual clinicians and doctors who treat women living with HIV. Outreach clinics were visited so that their clients could be invited to participate. Weekly reminder emails, showing the numbers of respondents recruited, were sent to the electronic mailing lists of women living with HIV to show progress, build a sense of owned momentum and encourage further outreach.

### Data analysis

The analysis of the survey responses started with the structured thematic headings. Quantitative data under each heading were examined to identify major trends. The qualitative free-text responses were read in relation to the key findings derived from the quantitative data. Two members of the core team coded responses to explore key themes in relation to the barriers to – and enablers for – achieving various desired outcomes in terms of sexual and reproductive health and human rights.

## Results

A full report of the study, including an executive summary, was made available online in January 2015 following a WHO Stakeholder Consultation in which the results of the online survey were presented.^4^ Some of the results of the study have also already been published in two peer-reviewed articles – on the impacts of gender-based violence^13^ and poor mental health^14^ on the sexual and reproductive health of women living with HIV – and other analyses of the survey data are in preparation. Here we focus on the methods, approach and main findings. The study covered 945 women living with HIV: 113 from the focus group discussions (Table 1) and 832 from the online survey (Table 2). These women came from 94 countries and were aged 15–72 years.^4^ The key issues raised by the community are summarized in Table 3.

**Table 2:** Numbers of respondents to the online survey of women living with HIV in 94 countries, 2014

Language	No. of respondents	No. of respondents who were women living with HIV
English	568	480
Russian	135	99
Spanish	128	104
Chinese	80	57
French	46	42
Portuguese	44	28
Bahasa Indonesian	27	22
**Total**	**1038**	**832**

HIV: human immunodeficiency virus.

**Table 3:** Key responses of the women living with HIV who participated in the online survey covering 94 countries, 2014

Response	No. of women answering relevant question	No. of women answering yes (%)**^a^**
Had experienced violence or fear of violence^b^	480	427 (89.0)
Always or usually had a healthy libido and/or feeling of sexual desire	479	154 (32.2)
Found service providers to be well trained, knowledgeable, friendly and supportive	589	297 (50.4)
Had been supported by service providers to make fertility-related choices	318	169 (53.1)
Had an unplanned pregnancy	312	177 (56.7)
Had accessed family-planning counselling	273	122 (44.7)
Had received advice on safe conception	314	227 (72.3)
Had received practical support on safe conception	304	168 (55.3)
Had a mental health issue after the diagnosis:	832	489 (58.8)
Depression	486	360 (74.0)
Shame	459	325 (70.8)
Self-blame	478	334 (70.0)
Feelings of rejection	468	327 (69.9)
Insomnia	459	314 (68.4)
Other^c^	473	285 (60.3)

^a^ Of the women answering the relevant question.

^b^ Before, since and/or because of positivity for human immunodeficiency virus.

^c^ Anxiety, body-image problems, loneliness/isolation and/or very low self-esteem.

Of the 480 participants in the online survey who answered the optional question on gender-based violence, only 53 (11.0%) reported never having experienced such violence. According to the respondents, gender-based violence from intimate partners increased after the diagnosis of the women’s HIV positivity. Gender-based violence from family members – other than intimate partners – and community members were often reported to start only after the women had received the diagnosis. Of the 489 women who answered some or all of the optional questions on mental health in the online survey, most reported that they had suffered the symptoms of depression and rejection after they received their diagnosis (Table 3) but only 98 (20.0%) claimed to have suffered mental health issues before the diagnosis. The respondents recommended that policy-makers and clinicians should address the issues of gender-based violence and poor mental health as part of a comprehensive global package of care for women living with HIV. The findings from the focus group discussions echoed and expanded upon these findings from the online survey.

In general, the women living with HIV who responded to the online survey appeared to trust the data recorders – as reflected in the large numbers of respondents and the positive comments received (Box 1) – and had a strongly-felt desire to inform the relevant policy-makers of their visions and challenges. Some participants revealed that they had shared experiences – either in the online survey or the focus group discussions – that they had never revealed to anyone before.

Box 1 **Selected quotes recorded during the online survey and focus group discussions of women living with HIV**

**From a member of the global reference group**

“Thank you to those of you who have sent others and me words of support in solidarity – your sisterhood is so appreciated! Sometimes it feels like life is unrelenting but I take comfort in knowing that all things, no matter how hard, pass. This is what keeps me going. Thanks for sharing what you are going through and have gone through – it takes such huge courage to share our own hurt and it is so hard. I have no words of wisdom but just sending you so much love and huge amounts of respect. I feel so privileged to know all of you. Thank you! Thank you!”

**From a survey respondent in the United States of America**

“I found the experience to be cathartic, much to my surprise. Surveys don’t generally have that effect. It was meaningful to answer questions that truly reflected my experiences both as a girl and young woman before HIV and since my diagnosis. Even though there were questions about violence and trauma that could have felt difficult, the fact that the survey was written by and for women living with HIV and in a tone that is empowering rather than victimizing, made my participation feel good and made me feel that I could be really honest in my answers.”

**From a facilitator of the focus group discussion in Myanmar**

“[The discussion participants] became active and spoke out, though they dared not speak at first. I shared my experiences and feelings so that they would open up their feelings. When we discussed about discrimination, they said there was no discrimination before their friends knew [that they had HIV infection]. But after discovering that they had the infection, their friends discriminated [against them] by not playing with them. One participant said that her friend’s aunty did not let her friend play with her. Another told how her friend stopped playing with her after discovering that her mother had died of AIDS. Then I asked, who comforts you when you feel bad, and two of them said that only their grandmother comforts because both of their parents had passed away.”

**From a survey respondent in Australia**

“We are not a one-size-fits-all [community]. Guidelines need to be responsive to individual needs and circumstances, they somehow need to take into account psychosocial factors that impact on my whole-of-life experience and not just my HIV.”

AIDS: acquired immunodeficiency syndrome; HIV: human immunodeficiency virus.

Participants responded positively to the online survey both as a reflective, therapeutic process and as an advocacy and learning tool. In both the online survey and focus group discussions, participants called for women living with HIV – in all of their diversity and at all of their different life stages – to be at the centre of all decision-making that affects their lives, so that all of their human and sexual and reproductive health rights can be realized. They called for clinical approaches to be underpinned by a strong human-rights framework, with laws that actively promote and protect human rights and gender equality instead of simply addressing violations of such rights and equality.

## Discussion

Here we describe the process of hearing the voices of the community of women living with HIV before the start of the process of developing guidelines. The approach used – of interweaving issues relating to sexual and reproductive health and human rights across the lifespans and diversities of women with HIV around the world – appears to have been welcomed among participants. Although such community participation has been supported elsewhere,^15^^,^^16^ here the values and preferences of the key affected population have been elicited for the first time at the start of guideline development. Although such an approach may not always be possible in other contexts or populations, it would merit replication, where possible, in the development of guidelines for many other health considerations. The online survey also provided an opportunity to strengthen institutional capacity and to build understanding among women living with HIV of policy and guideline development.

Women living with HIV often suffer from gender-based violence^13^ and poor mental health,^14^ and these problems may be exacerbated following disclosure of the woman’s HIV status.^17^ The outcomes highlight how the lives of women living with HIV are complex^4^ – partly as the result of interconnections between issues that often extend beyond the women’s sexual and reproductive health needs. For example, financial insecurity was not only seen as a substantial barrier to obtaining the care needed to protect the women’s sexual and reproductive health, but was also interwoven with the women’s decision-making about having children, more children or no children.

The study had several limitations. The prevalence we recorded was self-reported and, for most sections of the online survey, participants could choose whether to answer or ignore the questions. We have no way of knowing if the women who answered a question were representative of all the participants in the survey. For example, we cannot tell if, compared with the other participants, those with poor mental health or a history of poor mental health were more likely to answer the questions on mental health – perhaps because they were more interested in the topic – or less likely – perhaps because they were too embarrassed to admit they had mental health issues. More rigorous research, with direct observation or other forms of validation, is required. There is also some uncertainty about the quality and accuracy of the electronically translated responses – although most non-English responses were translated, unaided, by a professional translator. There may also have been selection bias, as most of the women engaging with the survey had access to support networks or groups and/or were advocates. In general, as the participants noted, the survey results are liable to underestimate the impacts of living with HIV, especially in terms of poverty, human rights abuses and violence. Policy-makers need to be more understanding of the complexity of the lives of women living with HIV and the interconnectedness of their problems. All women – including those living with HIV – need humane, holistic services that take into account the different stages of a woman’s life.

Although we found the survey results and the respondent’s attitudes to the survey approach encouraging, the future impact of the survey process and results and the cost–effectiveness of this approach remain unknown. However, in 2015, in response to demand from policy-makers and stakeholders in Latin America, the survey report was translated into Spanish and the survey results were presented in a web conference in Spanish.^18^^,^^19^Furthermore, in December 2014, the Positive Women charitable organization in Ukraine launched a nationwide survey – of the sexual and reproductive rights of women living with HIV in Ukraine – using the same survey tool as used in this study (S Moroz, personal communication, 2015).

The results from the global study reported here should be very useful in the development of new guidelines on the sexual and reproductive health and human rights of women living with HIV and should play an important role in facilitating evidence-based recommendations. Many of the respondents included in our online survey called for further research into (i) the sustained and meaningful engagement of the populations that are likely to be affected by new guidelines in the whole development process for such guidelines and (ii) the benefits and unintended consequences of such engagement.
